# A rare missense variant in *NR1H4* associates with lower cholesterol levels

**DOI:** 10.1038/s42003-018-0015-9

**Published:** 2018-02-08

**Authors:** Aimee M. Deaton, Patrick Sulem, Paul Nioi, Stefania Benonisdottir, Lucas D. Ward, Olafur B. Davidsson, Socheata Lao, Anna Helgadottir, Fan Fan, Brynjar O. Jensson, Gudmundur L. Norddahl, Aslaug Jonasdottir, Adalbjorg Jonasdottir, Asgeir Sigurdsson, Ragnar P. Kristjansson, Asmundur Oddsson, Gudny A. Arnadottir, Hakon Jonsson, Isleifur Olafsson, Gudmundur I. Eyjolfsson, Olof Sigurdardottir, Einar S. Bjornsson, Sigurdur Olafsson, Thora Steingrimsdottir, Thorunn Rafnar, Gudmundur Thorgeirsson, Gisli Masson, Gudmar Thorleifsson, Daniel F. Gudbjartsson, Hilma Holm, Unnur Thorsteinsdottir, Kari Stefansson

**Affiliations:** 1 deCODE Genetics/Amgen, Inc., Reykjavik, 101 Iceland; 2 Faculty of Medicine, University of Iceland, Reykjavik, 101 Iceland; 3 Department of Clinical Biochemistry, Landspitali University Hospital, Reykjavik, 101 Iceland; 4 Laboratory in Mjódd (RAM), Reykjavik, 109 Iceland; 5 Department of Clinical Biochemistry, Akureyri Hospital, Akureyri, 600 Iceland; 6 Department of Medicine, Landspitali University Hospital, Reykjavik, 101 Iceland; 7 Department of Obstetrics and Gynecology, Landspitali University Hospital, Reykjavik, 101 Iceland; 8 Division of Cardiology, Department of Internal Medicine, Landspitali University Hospital, Reykjavik, 101 Iceland; 9 School of Engineering and Natural Sciences, University of Iceland, Reykjavik, 101 Iceland

## Abstract

Searching for novel sequence variants associated with cholesterol levels is of particular interest due to the causative role of non-HDL cholesterol levels in cardiovascular disease. Through whole-genome sequencing of 15,220 Icelanders and imputation of the variants identified, we discovered a rare missense variant in *NR1H4* (R436H) associating with lower levels of total cholesterol (effect = −0.47 standard deviations or −0.55 mmol L^−1^, *p* = 4.21 × 10^−10^, *N* = 150,211). Importantly, *NR1H4* R436H also associates with lower levels of non-HDL cholesterol and, consistent with this, protects against coronary artery disease. *NR1H4* encodes FXR that regulates bile acid homeostasis, however, we do not detect a significant association between R436H and biological markers of liver function. Transcriptional profiling of hepatocytes carrying R436H shows that it is not a loss-of-function variant. Rather, we observe changes in gene expression compatible with effects on lipids. These findings highlight the role of FXR in regulation of cholesterol levels in humans.

## Abstract (web summary)

Aimee Deaton et al. identify a rare missense variant in the bile acid receptor gene *NR1H4*, which is associated with lower levels of total cholesterol in the Icelandic population. Hepatocytes expressing the missense variant showed altered expression of a small number of genes, with enrichment in lipid-related pathways.

## Introduction

Genetic studies have led to the discoveries of a large number of loci associated with lipid traits. These range from candidate gene studies yielding mutations causing Mendelian disorders such as familial hypercholesteremia^1–4^, to using genome-wide association studies (GWAS) to identify both common and low frequency sequence variants associating with lipid levels^5–7^.

Whole-genome sequencing of large numbers of people and imputation of detected variants into larger sets of genotyped individuals now provides the opportunity to discover new associations between rare variants and lipid traits.

We previously used this approach to discover 13 rare and low frequency variants with large effects on lipids^8^. Identifying variants associated with non High-Density Lipoprotein (non-HDL) cholesterol levels is of particular interest because of how they affect cardiovascular disease risk^9–11^. We recently reported rare loss-of-function mutations in *ASGR1* and *HP* associating with non-HDL levels and the risk of coronary artery disease^12,13^.

In this study, we searched for additional rare coding variants associating with cholesterol levels at previously unreported loci using a greatly expanded set of Icelanders who have had their genome sequenced (15,220 compared to 2,636) and/or been chip-genotyped (151,677 compared to 104,220). We discovered a novel association of a rare missense variant in *NR1H4* (encoding the bile acid receptor FXR) with levels of total and non-HDL cholesterol.

## Results

### A rare missense variant in NR1H4 associates with cholesterol levels

To search for novel rare variants associating with cholesterol levels, we sequenced the genomes of 15,220 Icelanders (34× coverage). We imputed variants detected in these data into 151,677 chip-genotyped individuals through long-range haplotype phasing and, additionally, calculated genotype probabilities for first-degree and second-degree relatives of those chip-typed^14,15^. This approach allowed us to test variants with minor allele frequencies as low as 0.03% (present in around 1 in 2000 people). We tested protein-coding variants for association with cholesterol levels (126,220 to 150,211 individuals screened; see Supplementary Table 1) using previously established methodology to determine genome-wide significance thresholds for different variant classes based on the number of markers tested per class^16^. We considered a *p*-value below 5 × 10^−8^ significant for missense variants and a *p*-value below 2.6 × 10^−7^ significant for loss-of-function mutations.

**Fig. 1:**
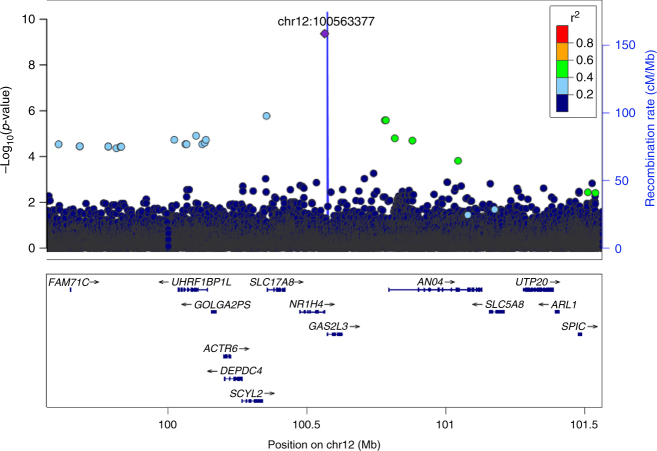
A missense variant in *NR1H4* associates with total cholesterol levels in Iceland. Association results for total cholesterol levels (*N* = 150,211) of variants in the region chr12:99563377−101563377. The purple diamond represents the variant resulting in the R436H missense mutation in *NR1H4* (located at chr12: 100563377). Other variants are colored according to correlation (*R*^2^) with this marker (legend at top-right). No marker in the region has an *R*^2^ of >0.53 with the leading marker. RefSeq genes in the region are displayed

We discovered a novel association between a missense variant in *NR1H4*, encoding the bile acid receptor FXR, and levels of total cholesterol at a genome-wide significant level (*p* = 4.21 × 10^−10^; Fig. 1). This variant is present in ~1 in 450 Icelanders (minor allele frequency = 0.11%; imputation information = 0.96) and results in an arginine to histidine substitution at amino acid 436 of the FXR protein (R436H; NCBI protein sequence, NP_005114.1:p.Arg436His, rs750672942_A). Throughout the text, we use R436H to refer to this variant, named according to the *NR1H4* transcript most highly expressed in liver (GTEx Portal^17^). Supplementary Table 2 shows the effect of the mutation on other *NR1H4* isoforms. We observed no homozygous carriers of R436H among 151,677 imputed Icelanders consistent with its low allele frequency. The *NR1H4* R436H variant has been observed at very low frequency in South Asian and Latino populations, five copies observed in 32,182 individuals in these populations, ten times lower frequency than in Iceland. The variant is absent from any of the 55,847 Europeans in the Genome Aggregation Database (GNOMAD, accessed 21st June 2017). In addition, R436H is absent from the UK10K data, (ALSPAC *N* = 1928 and Twin study *N* = 1854; European Aggregation Variation (EVA), accessed 18th December 2017) and is not tested by UK Biobank. It is also absent from 1000 genomes phase 3.

*NR1H4* R436H associates with lower total cholesterol levels than the reference allele (−0.55 mmol L^−1^, 95% confidence interval (CI) = −0.72 to −0.37 mmol L^−1^; effect = −0.47 SD; *p* = 4.21 × 10^−10^) (Table 1). No variant in the 2 Mb region has an *R*^2^ >0.5 with this variant (Fig. 1) and no other missense variant in *NR1H4* associates with cholesterol levels (three other variants tested, *p* ≥ 0.12; Supplementary Table 3). After conditioning on *NR1H4* R436H, no variant in the 500 kb region around this variant has an association with total cholesterol levels (*p* > 1.3 × 10^−4^ for all 3677 markers tested). These data suggest that R436H is the variant affecting cholesterol levels. *NR1H4* is highly expressed in liver and has a role in lipid metabolism^18^, whereas none of the other protein-coding genes in the region has known biological links with cholesterol^19,20^, further supporting a role for *NR1H4* in regulation of cholesterol levels.

**Table 1:** Association of *NR1H4* R436H with lipid traits in Iceland

Trait	Effect (95% CI)		*p*-value	Value in population (mmol L^-1^)	*N* measured
	mmol L^-1^ estimate	SD estimate			
Total cholesterol	−0.55 (−0.72,−0.37)	−0.47 (−0.62, −0.32)	4.21 × 10^−10^	5.33 ± 1.168	150,211
Non-HDL cholesterol	−0.50 (−0.69,−0.31)	−0.43 (−0.59, −0.27)	1.36 × 10^−7^	4.948 ± 1.166	136,326
LDL cholesterol	−0.45 (−0.62,−0.27)	−0.42 (−0.59, −0.26)	3.46 × 10^−7^	3.327 ± 1.053	126,220
HDL cholesterol	−0.06 (−0.13,0.02)	−0.13 (−0.29, 0.04)	0.13	1.425 ± 0.441	136,736
	**% change**	**SD estimate**			
Triglycerides	7.07 (−15.13,0.65)	−0.14 (−0.30, 0.02)	0.090	1.24 (0.73, 2.09)	119,624

Effect sizes are shown in millimoles per litre (mmol L^−1^) or percentage change as well as in standard deviations (SD); values in brackets represent the 95% confidence interval (CI). Lipid measurements and calculation of non-HDL and LDL cholesterol are described in the methods. For each lipid trait, the mean value and standard deviation in the population are given. As triglycerides have a log-normal distribution, the population mean, and standard deviation were calculated for log-transformed values and then transformed back to original units. To convert the values for total, non-HDL, LDL, and HDL cholesterol to mg dL^−1^ multiply by 38.67. To convert the values for triglycerides to mg dL^−1^ multiply by 88.57

A substantial fraction of the total cholesterol lowering effect of R436H is through its effect on non-HDL cholesterol levels (−0.50 mmol L^−1^, 95% CI = −0.69 to −0.31 mmol L^−1^; effect = −0.43 SD; *p* = 1.36 × 10^−7^) (Table 1). This effect is of a similar magnitude to that of variants in *PCSK9* (Arg46Leu, effect = −0.48 SD) and *APOE* (Arg176Cys, effect = −0.47 SD) that have well-established effects on lipid levels^21,22^. R436H also associates with LDL cholesterol levels (−0.45 mmol L^−1^, 95% CI = −0.62 to −0.27 mmol L^−1^; *p* = 3.46 × 10^−7^) (Table 1). We did not detect a significant association between R436H and levels of HDL cholesterol (−0.06 mmol L^−1^, 95% CI = −0.13 to 0.02 mmol L^−1^; *p* = 0.13) or triglycerides (7.07% change, 95% CI = −15.13% to 0.65%; *p* = 0.09) (Table 1).

Sequence variants altering non-HDL cholesterol levels most often affect risk of coronary artery disease proportionally to their effect on non-HDL cholesterol^8,23^ although there are exceptions to that^12,24^. We therefore assessed the effect of R436H on coronary artery disease and myocardial infarction and observed effects consistent with those predicted from the non-HDL cholesterol effect of other genetic variants (i.e., lower non-HDL cholesterol, lower disease risk) (Table 2; Supplementary Fig. 1; Supplementary Table 3). Carriers of *NR1H4* R436H tend to develop coronary artery disease later (5.2 years, 95% CI = 1.4–9.1 years; *p* = 0.0078) and myocardial infarction later (6.4 years, 95% CI = 1.7–11 years, *p* = 0.0069) than non-carriers (Table 2). As expected from the conferred protection against coronary artery disease, carriers of R436H have a lifespan that is, on average, 1.9 years longer than that of non-carriers (95% CI = 0.2–3.5 years; *p* = 0.031) (Table 2).

**Table 2:** Association of *NR1H4* R436H with cardiovascular disease in Iceland

Disease	OR (95% CI)	*p*-value	*N* (cases/controls)
Myocardial infarction at or before age 75	0.49 (0.27, 0.87)	0.014	16,256/319,516
Myocardial infarction all	0.68 (0.43, 1.07)	0.092	23,965/311,807
Coronary artery at or before age 75	0.61 (0.38, 0.97)	0.039	25,544/328,262
Coronary artery disease all	0.72 (0.48, 1.07)	0.103	37,782/318,845
**Trait**	**Effect in years (95% CI)**	***p***-**value**	***N*** **measured**
Coronary artery disease age of diagnosis	5.2 (1.4, 9.1)	0.0078	37,831
Myocardial infarction age of onset	6.4 (1.7, 11)	0.0069	23,995
Lifespan (after age 50)	1.9 (0.2, 3.5)	0.031	119,767

Myocardial infarction and coronary artery disease cases and controls were selected as described in the methods and the effect on disease risk is shown as an odds ratio (OR) with 95% confidence interval (CI). For age of coronary artery disease diagnosis, age of myocardial infarction onset, and lifespan, effect sizes are shown in years with 95% CI

### NR1H4 R436H does not associate with measures of liver function

*NR1H4* encodes FXR, the nuclear farnesoid X-receptor, whose best-characterized function is in maintaining bile acid homeostasis^18^. We therefore examined whether *NR1H4* R436H associates with measures of hepatobiliary function using a large collection of clinical chemistry results (*N* = 92,163–172,086). R436H does not significantly associate with levels of biological markers widely used as tests of liver function i.e., alkaline phosphatase, alanine transaminase, aspartate transaminase, bilirubin, gamma glutamyl transpeptidase, or albumin (*p* ≥ 0.22 for all six associations; Supplementary Table 5). Furthermore, we did not detect an association between R436H and the risk of gallstones for which we have 8447 cases. The low frequency of the variant means that there is a large confidence interval for the estimated effect on disease risk (OR = 1.01, 95% CI = 0.58–1.76; p = 0.96) and thus limited power to detect a small effect. We have 80% power to detect association of R436H with gallstones for OR = 1.73 (Supplementary Table 6).

We also tested R436H for association with additional phenotypes in our database but did not find any association with disease (*p* < 0.001; 5742 phenotypes tested) or quantitative traits (*p* < 1 × 10^−4^; 8914 phenotypes tested). No variants near *NR1H4* have been reported to associate with metabolic traits (GWAS catalog accessed 21st June 2017)^25^. The only reported GWAS hit near *NR1H4* is the common variant rs12296850 at *SLC17A8* (100 kb away; EAF = 76%) associating with squamous cell carcinoma of the lung in Chinese individuals (*p* = 1 × 10^−10^)^26^. This variant is not correlated with R436H (*R*^2^ < 0.01).

Biallelic loss-of-function variants in *NR1H4* (p.Arg176*, p.Tyr139_Asn140insLys and a 31.7 kb deletion spanning the first two coding exons) have been reported to associate with progressive familial intrahepatic cholestasis (PFIC) characterized by liver dysfunction, elevated aminotransferases, hyperbilirubinemia, and elevated prothrombin time^27^. We discovered a very rare predicted loss-of-function mutation in *NR1H4* affecting a splice donor (NM_005123.3:c.817_819+2delATTGT; Minor Allele Frequency  = 0.016%) (Supplementary Note; Supplementary Table 7), which did not associate with hepatic dysfunction in heterozygotes, although we are only powered to detect large effects. No homozygous carriers of this mutation were found in Iceland. The *NR1H4* splice donor mutation is not significantly associated with levels of total or non-HDL cholesterol although weak effects cannot be excluded (Supplementary Note; Supplementary Tables 8–10).

### Investigating the impact of R436H on NR1H4 in cell culture models

FXR acts as a heterodimer with the retinoid X-receptor (RXR) to regulate transcription in response to bile acids. Although FXR’s best-characterized role is in regulating bile acid synthesis and transport, it also has roles in lipid metabolism, glucose metabolism, and autophagy^18,28^.

**Fig. 2:**
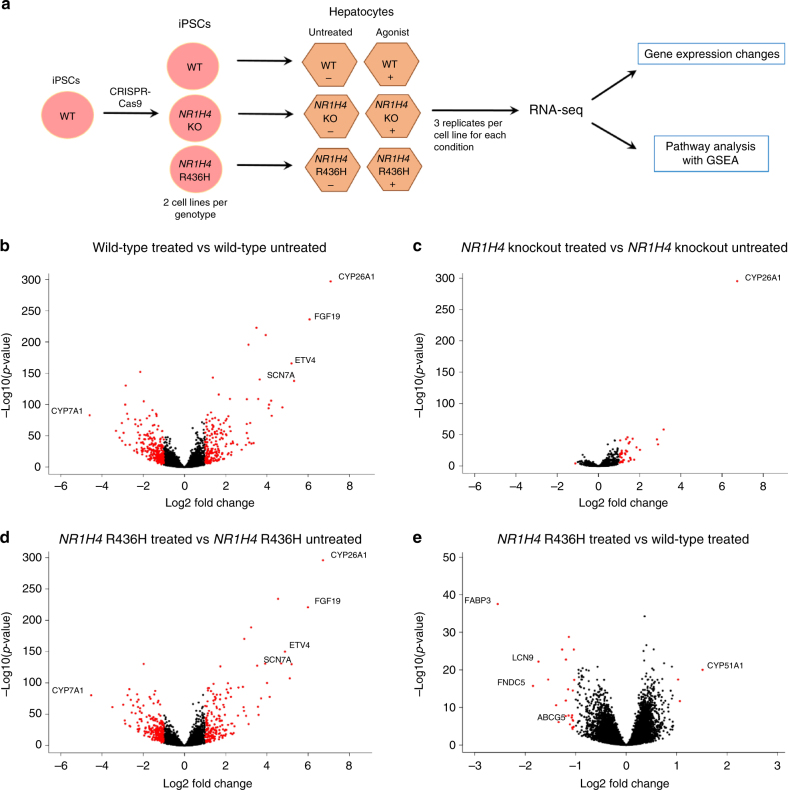
FXR agonist treatment followed by RNA-seq on hepatocytes with *NR1H4* mutations. **a** Experimental outline. iPSCs lines carrying homozygous *NR1H4* mutations were engineered using CRISPR-Cas9 and differentiated into hepatocytes. Hepatocytes were treated with FXR agonist GW4064 (“Agonist”+) or DMSO (“Untreated”−) for 24 h and harvested for RNA-seq. For each condition, 6 replicates were generated (3 replicates from each of two independent cell lines—see Methods). **b**–**e** Gene expression differences in iPSC-derived hepatocytes. **b** Wild-type cells treated with agonist vs. untreated cells. **c** *NR1H4* knockout cells treated with agonist vs. untreated cells. Induction of *CYP26A1* is likely to be a response of hepatocytes to drug exposure. **d** *NR1H4* R436H cells treated with agonist vs. untreated cells. **e** *NR1H4* R436H agonist-treated hepatocytes vs. wild-type agonist-treated hepatocytes. For all panels, the *x*-axis shows log_2_-fold change in gene expression; genes to the right are upregulated and genes to the left are downregulated. The *y*-axis shows −log10 of the adjusted *p*-value. Fold-change and significance were calculated using DESeq2, which uses Benjamini–Hochberg correction for *p*-value adjustment. Each Entrez gene is represented by a dot and red dots mark genes showing a log_2_-fold change in expression >1 with *p*-adjusted <10^−3^. Selected genes are labeled. Aimee Deaton et al. identify a rare missense variant in the bile acid receptor gene *NR1H4*, which is associated with lower levels of total cholesterol in the Icelandic population. Hepatocytes expressing the missense variant showed altered expression of a small number of genes, with enrichment in lipid-related pathways

To examine the effect of the *NR1H4* R436H variant on NR1H4/FXR function, we used various cell culture models. We started by assessing the ability of R436H to activate transcription of a luciferase reporter containing the canonical FXR response element by overexpressing either wild-type FXR or FXR R436H along with the reporter in HepG2 cells. FXR R436H activated expression of the luciferase reporter to the same level as the wild-type protein upon treatment with an FXR agonist, suggesting that it does not disrupt FXR transcriptional function (Supplementary Fig. 2). To explore the effects of R436H in a more endogenous context we used CRISPR-Cas9 genome editing to generate homozygous *NR1H4* R436H and *NR1H4* knockout human iPSC lines (Supplementary Fig. 3). As *NR1H4* is most highly expressed in the liver, we then differentiated wild-type and mutant iPSCs to hepatocyte-like cells (hereafter described as hepatocytes). As FXR is a transcription factor, we used gene expression as a means of examining the impact of the R436H variant on the protein’s function. To examine this, hepatocytes were treated with an FXR agonist for 24 h, RNA was then extracted from the cells, and transcript abundance measured by RNA-seq (Fig. 2a).

The global response to FXR activation was maintained in *NR1H4* R436H hepatocytes. In wild-type and R436H hepatocytes, out of 11,720 genes examined, 442 and 415 genes, respectively, changed expression upon agonist treatment (log_2_-fold change >1, adjusted *p*-value (Benjamini–Hochberg correction) < 10^−3^) and the vast majority of these were overlapping (362 genes that changed in R436H upon agonist treatment also changed in wild-type cells) (Fig. 2b, d; Supplementary Data 1). These genes included both well-characterized FXR-regulated genes, such as *CYP7A1*, *FGF19*, and *NR0B2*, and novel FXR targets, for example *ETV4* and *SCN7A*. In contrast, *NR1H4* knockout hepatocytes largely failed to respond to FXR activation with just 39 genes changing expression using the same criteria (Fig. 2c; Supplementary Data 1). These data confirm our findings from the reporter gene assay that R436H does not result in a loss of FXR function.

We next investigated the differences between the transcriptomes of wild-type and R436H hepatocytes. We found a greater number of gene expression differences between these two cell lines when we compared them in the FXR agonist-treated state than in the untreated state (28 vs. 15 genes, log_2_-fold change >1, adjusted *p*-value <10^−3^; Supplementary Data 1). The majority of the 28 genes that showed a difference in expression between wild-type and R436H cells in the agonist-treated state demonstrated less expression in R436H cells than in wild-type cells (Fig. 2e; Supplementary Data 1). Nine of these 28 genes change expression in response to FXR agonist treatment in wild-type cells, and for eight of these genes this expression response is abolished or decreased in R436H cells. Half of the 28 genes overlap with changes seen in *NR1H4* knockout compared to wild-type agonist-treated hepatocytes (Supplementary Data 1) indicating disruption of FXR regulation for this small subset of genes. Several of the genes differentially expressed in R436H agonist-treated hepatocytes have roles in lipid biology. The largest change was downregulation of *FABP3*, which encodes a protein involved in fatty acid transport^29,30^. Other notable genes were *ABCG5*, which encodes a lipid transporter^31^ and shows repression upon agonist treatment in R436H cells but not in wild-type cells, as well as *CYP51A1*, which encodes an enzyme involved in cholesterol biosynthesis^32^ (Table 3).

**Table 3:** Selected genes with differential expression in *NR1H4* R436H agonist-treated cells compared to wild-type agonist-treated cells

Gene	Log_2_-fold change (absolute change)	Adjusted *p*-value	Max FPKM	FXR binding (*N* = 1066)	Change in WT agonist-treated vs. untreated (*N* = 442; log_2_-fold change > 1, adjusted *p*-value < 10^−3^)	Change in R436H agonist-treated vs. untreated (*N* = 415; log_2_-fold change > 1, adjusted *p*-value < 10^−3^)	Change in KO agonist-treated vs. WT treated (*N* = 322; log_2_-fold change > 1, adjusted *p*-value < 10^−3^)	Lipid-related pathway
*FABP3*	−2.54 (−5.8)	2.89 × 10^−38^	17.8	Yes	Up	—	Down	PPAR signaling (KEGG)
*ABCG5*	−1.05 (−2.1)	3.43 × 10^−7^	3.2	Yes	—	Down	—	Metabolism of lipids and lipoproteins (reactome); lipid digestion, mobilization and transport (reactome)
*CYP51A1*	1.51 (2.8)	9.14 × 10^−21^	1.3	—	—	—	Up	Metabolism of lipids and lipoproteins (reactome); cholesterol biosynthesis (reactome)

Differential expression was assessed using DESeq2 and genes with log_2_-fold change > 1 (absolute change > 2-fold) and adjusted *p*-value < 10^−3^ (Benjamini–Hochberg correction) were considered differentially expressed. Overall, 28 genes showed differential expression in R436H agonist-treated cells compared to wild-type agonist-treated cells. Data on selected genes are shown in the table. The maximum fragments per kilobase million seen in any condition (Max FPKM) is included to give a measure of the gene’s expression level. FXR binding in primary human hepatocytes^51^ is included for each gene. If the gene also changes expression in wild-type (WT) agonist-treated cells compared to WT-untreated cells, R436H agonist-treated cells compared to R436H-untreated cells or knockout (KO) agonist-treated cells compared to WT agonist-treated cells, the direction of expression change is shown. Selected lipid-related pathways (reactome or KEGG) to which each gene belongs are displayed

Gene set enrichment analysis^33,34^ revealed that pathways connected to lipids showed significant enrichment (FDR < 5%) for genes upregulated in R436H agonist-treated cells compared to wild-type agonist-treated cells. These include pathways for cholesterol biosynthesis, phospholipid metabolism, lipid metabolism, and valine, leucine and isoleucine degradation (which contains a number of genes connected to cholesterol metabolism). Sphingolipid metabolism pathways were also upregulated (Supplementary Table 11; Supplementary Data 2). Many of these lipid-related pathways were also enriched for genes upregulated in *NR1H4* knockout cells compared to wild-type cells (Supplementary Data 2). Thus, while the general function of FXR is maintained in *NR1H4* R436H cells, there are subtle changes in the expression of genes with connections to lipids.

R436H is located in the dimerization interface of FXR that interacts with RXR and does not affect amino acids involved in DNA binding, ligand binding, or co-activator interaction (Supplementary Fig. 4A). Modeling using the published FXR crystal structure (PDB ID: 4OIV) suggested that R436H might change the protein surface charge at the dimerization interface from positive to weakly negative (Supplementary Fig. 4B, C). The corresponding dimerization interface on RXR is positively charged (Supplementary Fig. 4D) and one possibility is that the negative FXR surface charge introduced by R436H could better facilitate dimerization between FXR and RXR.

Our data are not consistent with *NR1H4* R436H being a complete loss-of-function mutation but suggest that the variant somehow alters the activity of FXR.

## Discussion

In this study, we discovered associations between the rare missense variant R436H in *NR1H4* and lower levels of both non-HDL and total cholesterol. As sequence variants that affect non-HDL cholesterol often have an effect on the risk of coronary artery disease and myocardial infarction that is proportional to the effect on non-HDL cholesterol, we evaluated the association of R436H with the risk and age of onset of these diseases. As R436H is rare, our estimates of its effect are not precise, although the associations are significant and consistent with the effect of R436H on non-HDL cholesterol. Similarly, carriers of R436H have an increased life expectancy consistent with the effect of the variant on atherosclerotic diseases. R436H is absent or vanishingly rare in non-Icelandic populations, which is not unexpected given its low frequency in Iceland. This means that it is currently not possible to attempt replication of the association of R436H with cholesterol levels in a separate study group.

In addition to its well-characterized role in regulating bile acid homeostasis, FXR is involved in lipid metabolism^18,35,36^. However, FXR-dependent regulation of non-HDL cholesterol levels in humans is not well-established. We note that *Nr1h4* homozygous knockout mice have increased cholesterol levels, opposite to what we observed for carriers of *NR1H4* R436H, further supporting our data showing that R436H is not a loss-of-function mutation. In addition, *NR1H4* knockout mice have changes in HDL cholesterol and triglycerides not seen in R436H carriers^19,35^ (http://www.mousephenotype.org/data/genes/MGI:1352464).

Compatible with the role of FXR in bile acid homeostasis, biallelic loss-of-function variants in *NR1H4* have been reported to cause progressive familial intrahepatic cholestasis^27^. One allele of *NR1H4* R436H, described here, does not associate with markers of liver function or gallstones in the Icelandic population suggesting that this variant might alter rather than completely abolish FXR function.

Consistent with this, modeling of *NR1H4* R436H in iPSC-derived hepatocytes showed that it is not a complete loss-of-function mutation as the global response to FXR activation in these cells is very similar to that of wild-type cells. However, we did observe subtle gene expression differences compatible with an effect on lipids when we compared R436H agonist-treated hepatocytes to wild-type. Of the genes that do change expression in R436H hepatocytes, half overlap with changes seen in *NR1H4* knockout hepatocytes. This suggests that R436H may disrupt FXR regulation of a small subset of genes. Gene set enrichment analysis, which does not impose a threshold for differential gene expression, shows that genes belonging to lipid pathways are upregulated in R436H agonist-treated cells compared to wild-type agonist-treated cells. These pathways include cholesterol biosynthesis, metabolism of lipids, and lipoproteins as well as sphingolipid metabolism. Sphingolipids have been reported to influence cholesterol trafficking and lipid metabolism^37,38^.

We hypothesize that gene expression changes due to R436H may be of greater magnitude in the context of the whole organism. We have modeled R436H in hepatocytes; however, FXR is expressed in both the liver and small intestine^18^. It is possible that R436H has an effect in both organs and/or that communication between the liver and intestine has role in altering cholesterol levels in humans. Examining FXR R436H in a gut–liver cell culture model is a compelling area for further study.

Protein surface charge modeling suggests that R436H enhances dimerization between FXR and RXR. Enhanced dimerization might alter the dynamics of FXR/RXR interaction with DNA or perhaps sequester RXR away from other dimerization partners involved in lipid regulation such as LXR and PPAR-alpha^39,40^. In this way, R436H might affect the expression of genes regulated by other RXR heterodimers as well as FXR/RXR target genes. We note that variants in *NR1H3*, which encodes LXR, have an association with HDL cholesterol levels^8,41^ and variants in the gene encoding PPAR-alpha are associated with total and LDL cholesterol levels^7^.

The precise mechanism by which R436H affects cholesterol levels in humans remains to be uncovered. Nonetheless, our findings highlight a role for FXR in the regulation of non-HDL cholesterol levels and hence the risk of cardiovascular diseases.

## Methods

### Icelandic study population

Study participants were enrolled as part of various genetics programs at deCODE. All genotyped individuals provided informed consent, and the study was approved by the Data Protection Commission of Iceland and the Icelandic National Bioethics Committee. We obtained blood lipid levels (total cholesterol, non-HDL cholesterol, LDL cholesterol, HDL cholesterol, and triglycerides) and levels of hepatobiliary markers (alkaline phosphatase, alanine transaminase, aspartate transaminase, bilirubin, gamma glutamyl transferase, and albumin) from three of the largest clinical laboratories in Iceland with measurements taken between 1990 and the end of 2015: (i) Landspitali—The National University Hospital of Iceland (LUH), (ii) The Icelandic Medical Center (Laeknasetrid) laboratory in Mjodd, Reykjavik, Iceland and (iii) Akureyri Hospital, The Regional Hospital in North Iceland, Akureyri, Iceland. Non-HDL cholesterol was calculated as total cholesterol minus HDL cholesterol. LDL cholesterol was calculated using the Friedewald equation for samples with triglyceride levels below 4.00 mmol L^−1^.

CAD and MI cases were defined as (i) individuals in the MONICA registry^42^ who suffered myocardial infarction before the age of 75 years in Iceland between 1981 and 2002; (ii) subjects with discharge diagnoses of CAD or MI (ICD-9 codes 410.*, 411.*, 412.*, and 414.* or ICD-10 codes I20.0, I21.*, I22.*, I23.*, I24.*, and I25.*) from LUH between 1987 and 2015; (iii) subjects diagnosed with significant angiographic CAD (at least 50% luminal reduction) identified from a nationwide clinical registry of coronary angiography and percutaneous coronary interventions at LUH between 1987 and 2012; (iv) subjects undergoing coronary artery bypass grafting (CABG) procedures at LUH between 2002 and 2015; or (v) individuals with cause of death or a contributing cause of death listed as myocardial infarction or CAD on death registries between 1996 and 2009. Coronary angiograms were evaluated by interventional cardiologists. The coronary artery disease sample set consisted of 37,782 cases including 25,075 cases with age at onset before 76 years of age and 5288 with early onset disease (occurring before age 50 for men and age 60 for women). The myocardial infarction sample set consisted of 23,965 cases including 16,256 cases with age at onset before 76 years of age and 3027 cases with early onset myocardial infarction (before age 50 for men and age 60 for women). Controls were participants in various genetic studies at deCODE Genetics without known cardiovascular disease.

Association testing was performed for other binary and quantitative traits in our database, which has been described previously^14,15^. This database includes extensive medical information on a variety of diseases and traits obtained through collaborations with medical specialists at LUH and other medical centers in Iceland.

### Whole-genome sequencing and Illumina single-nucleotide polymorphism chip genotyping

Genotyping of all samples was carried out at deCODE Genetics using previously described methods^14,15^. Whole-genome sequencing was performed for 15,220 Icelanders who were recruited as part of various genetic programs at deCODE Genetics, to a mean depth of at least 10× (median 34×) using Illumina technology. SNPs and indels in the whole-genome sequencing data were identified using the Genome Analysis Toolkit HaplotypeCaller^43^. These variants were then imputed into 151,677 Icelanders who had been genotyped with various Illumina SNP chips and their genotypes phased using long-range phasing. In addition, using the Icelandic genealogical database, genotype probabilities were calculated for first-degree and second-degree relatives of chip-genotyped individuals^14,15^. The effects of sequence variants on protein-coding genes were annotated using the Variant Effect Predictor (VEP)^44^ using protein-coding transcripts from RefSeq.

### Sanger sequencing and re-imputation

Twelve carriers of the *NR1H4* splice donor deletion were detected by whole-genome sequencing. We identified relatives of these carriers using the Icelandic genealogical database and performed Sanger sequencing on 94 potential carriers. In this set, we identified 47 carriers. We used this genotype data to re-impute the variant into the Icelandic data set. All carriers of this predicted loss-of-function variant in Iceland are heterozygous and no one carried both this variant and R436H.

### Association testing

Associations between imputed genotypes and serum lipids (non-HDL cholesterol, HDL cholesterol, LDL cholesterol, and triglycerides) and markers of hepatobiliary function (alkaline phosphatase, alanine transaminase, aspartate transaminase, bilirubin, gamma glutamyl transferase, and albumin) in the Icelandic data set were tested using a using a linear mixed model implemented in BOLT-LMM^45^, extended to analyze first and second-degree relatives of chip-genotyped individuals. Individuals were weighted according how informative the chip genotypes were about their genomes.

All measurements were adjusted for age and measurement site for each sex separately. The lipid measurements were further adjusted for statin use. After adjustment and inverse normal transformation we took an average value for each individual over all available measurements. Given their approximately log-normal distribution, triglyceride levels were log transformed prior to adjustment.

To obtain effect estimates in mmol L^−1^, the estimates from the regression analysis were multiplied by the estimated standard deviation for lipid levels in the population. As triglyceride levels were log-transformed, the corresponding effect estimates were calculated for the transformed data and are presented as percentage change.

Associations between variants and binary traits (myocardial infarction, coronary artery disease and gallstones) was tested using logistic regression treating disease status as the responses and the number of copies of the variant as the explanatory variable^14^.

We used LD score regression reference to scale the *χ*^2^ test statistics for both the quantitative trait and case control association tests^46^.

### Power calculations

Estimation of power entails the question of how likely we are to reject a null hypothesis of no effect, given the true effect and our sample. We assumed that the estimated effect of a variant on a given phenotype is $\hat \beta$ in Iceland and that σ is the standard error. To calculate the power to reject the null hypothesis of no association in Iceland, we have to assume a true effect for this variant, $\beta\, \ne\, 0$.

Assume that $\hat \beta |\beta \sim N\left( {\beta ,\sigma ^2} \right)$ and let $t_{\alpha /2}$ be the *z*-score threshold for having a two-sided *p*-value of $\alpha$, $\alpha = 2(1 - {\mathrm{\Phi }}\left( {t_{\alpha /2}} \right))$. The power to reject the null hypothesis of no association in Iceland given $\beta$ at the two-sided $\alpha$ level is:

$$p\left( { - t_{\frac{\alpha }{2}} > \frac{{\hat \beta }}{\sigma } > t_{\frac{\alpha }{2}}{\mathrm{|}}\beta } \right) = p\left( { - t_{\frac{\alpha }{2}} - \frac{\beta }{\sigma } > \frac{{\hat \beta - \beta }}{\sigma } > t_{\frac{\alpha }{2}} - \frac{\beta }{\sigma }{\mathrm{|}}\beta } \right) \\ = 1 - \left( {{\mathrm{\Phi }}\left( {t_{\frac{\alpha }{2}} - \frac{\beta }{\sigma }} \right) - {\mathrm{\Phi }}\left( { - t_{\frac{\alpha }{2}} - \frac{\beta }{\sigma }} \right)} \right)$$

For OR, $\beta$ = log(OR).

To calculate our power to detect the expected effect of R436H on coronary artery disease (CAD) risk given the non-HDL and CAD effect of other non-HDL associated variants, we performed regression for 108 reported non-HDL variants^23^ to examine the relationship between their effect on non-HDL cholesterol levels and risk of CAD at or before age 75. By fitting the best linear regression through the origin (Supplementary Fig. 1) we got the regression line: CAD(OR) = 1.0 + 0.45 × effect on non-HDL (mmol L^−1^). The observed non-HDL effect of R436H is −0.50 mmol L^−1^ and, given that effect, the expected CAD (OR) of R436H is 0.77. Assuming that the true effect of R436H on CAD risk is 0.77, the power to reject a null hypothesis of no effect at the two-sided significance level *α* = 0.05 is 0.19. We note that observed effect of R436H on CAD at or before 75 is OR = 0.61 with a 95% CI of (0.38, 0.97), which encompasses 0.77.

### Luciferase assays

An FXR reporter vector was constructed using the pLightSwitch_LR vector (Active motif) and contained three copies of a classical inverted repeat FXR response element upstream of a minimal TK promoter. The sequence inserted was caagAGGTCAtTGACCTttttcaagAGGTCAtTGACCTttttcaagAGGTCAtTGACCTtttt. Empty pLightSwitch_LR was used as a negative control.

Expression plasmids for wild-type, R436H and delAF2 FXR as well as RXR were obtained from Genscript. Details as follows:

FXR wild-type: pcDNA3.1 + DYK NM_005123

FXR R436H: pcDNA3.1 + DYK NM_005123 with R436H variant (CGC to CAG)

FXR delAF2: pcDNA3.1 + DYK NM_005123 with delAF2 (protein truncated at amino acid 462)

RXR-alpha: pcDNA3.1 + N-HA NM_002957

HepG2 cells were transfected in 96 well format with 100ng of luciferase reporter along with 25ng FXR and 25ng RXR expression plasmid using TransfeX (ATCC). Four replicates per condition were performed for each experiment. 5 µM GW4064 (Sigma Aldrich) or DMSO was added to cells 24 h after transfection. Luciferase assays were performed 24 h after the addition of GW4064 using LightSwitch Luciferase Assay reagent (Active Motif) according to manufacturer’s instructions and read using a Perkin-Elmer Envision plate reader.

### CRISPR-Cas9 editing of FXR variants in human iPSCs

Genome editing of a human episomal iPSC line (Thermofisher #A18945) was performed by Cellular Dynamics International (Madison, Wisconsin). Nucleases were designed to target the genome close to the desired modification areas (exon 4 of NM_005123 for *NR1H4* knockout, or codon R436 for *NR1H4* R436H). To generate the knockout lines, plasmid DNA encoding the exon 4 nuclease was electroporated into the starting iPSC line. At 7–10 days post-electroporation, single cell cloning was performed and DNA from the resulting clones was PCR amplified and sequenced to identify both heterozygous and homozygous frameshift indels. The PCR amplicon was then cloned into a plasmid and sequenced to confirm each individual allele. For R436H, plasmid DNA encoding the nuclease was electroporated into the starting iPSC line along with a 73 nucleotide single stranded oligonucleotide donor molecule (IDT, Coralville, IA) containing the R436H modification (as well as silent modifications at codons L437 and T438 to aid screening). At 7–10 days post-electroporation, single cell cloning was performed and the resulting clones were screened by allele-specific PCR for correct targeting. Heterozygous clones were re-targeted to generate homozygous lines.

Clones underwent a number of tests to verify correct targeting and the lack of unintended modifications. The *NR1H4* regions were amplified by PCR and characterized by Sanger sequencing to determine that one or both alleles were engineered as desired, and that no other insertions or deletions had been introduced on either the targeted or un-engineered alleles. Nucleases had been designed and chosen to avoid predicted off-target cutting within exons, within 5 kb of a transcriptional start site, within 5 kb of a stop codon, or in an intron within 500 bp of a stop codon. G-banding karyotyping was carried out (WiCell, Madison, WI) to confirm a normal chromosome complement. Lines were confirmed to be free of mycoplasma contamination. A proprietary gene expression panel showed that the cell lines were pluripotent. The absence of plasmid integration was confirmed by the lack of PCR amplification using primers containing plasmid DNA sequences.

Two independent homozygous *NR1H4* knockout iPSC lines (KO1 and KO2) and two independent homozygous R436H iPSC lines (R436H1 and R436H2) were used for all subsequent experiments.

### iPSC culture and hepatocyte differentiation

iPSCs were grown on matrigel-coated plates and maintained in Essential 8 Flex Medium (Invitrogen). Passaging was carried out using Versene (Invitrogen). Hepatocyte differentiation was based upon a protocol developed by Hannan and colleagues^47^. One day prior to the start of differentiation iPSCs were harvested using Accutase and plated at a density of 5–7.5 × 10^4^ cm^−2^ on matrigel-coated plates in mTeSR1 medium (Stem cell technology) and 3 μM rock inhibitor (Y-27632). Differentiation to definitive endoderm was performed using STEMdiff Definitive Endoderm Kit (Stem Cell Technologies) according to manufacturer’s instructions (days 1–4). Hepatic endoderm differentiation was achieved by treating cells with 10 ng mL^−1^ bFGF (Invitrogen) and 20 ng mL^−1^ BMP4 (R&D systems) in RPMI-1640 medium (Sigma Aldrich) containing B27 minus vitamin A (Invitrogen 12587010) (days 5–9). Cells were then differentiated into hepatoblasts in RPMI medium containing B27 (Invitrogen 17504044), 20 ng mL^−1^ HGF (R&D Systems), 2.5 µM Blebbistatin (Invitrogen), 50 ng mL^−1^ BMP4, 3 µM CHIR99021 (Biovision), 60 mg mL^−1^ FGF10 (R&D Systems), 10 ng mL^−1^ bFGF and 25 µg mL^−1^ Gentamicin (Invitrogen) (days 10–14). Hepatoblasts were treated with RPMI containing B27 minus vitamin A, 20 ng mL^−1^ Oncostatin M (R&D systems), 0.1 µM Dexamethasone (Sigma Aldrich), iCell Hepatocytes 2.0 medium supplement (Cellular Dynamics International), and 25 µg mL^−1^ Gentamicin for 5 days to achieve differentiation to hepatocyte-like cells (days 15–19). From day 20 of differentiation onwards cells were maintained in RPMI containing B27 minus vitamin A, 0.1 µM Dexamethasone, iCell Hepatocytes 2.0 medium supplement, and 25 µg mL^−1^ Gentamicin. Medium was changed daily throughout the differentiation process. We confirmed expression of the hepatocyte markers HNF4-alpha, ASGR1 and alpha-1 anti-trypsin on differentiated cells using flow cytometry.

At day 20 of differentiation either 5 µM of GW4064 (Sigma Aldrich) or DMSO was added to hepatocytes and cells were harvested for RNA-seq after 24 h of treatment.

For each genotype two cell lines were differentiated. In the case of mutant genotypes, these were two independent lines and for the parental wild-type line two separate passages of cells were used. For each of these cell lines, three biological replicates were differentiated for each treatment condition, giving a total of six replicates per sample. Replicate number was chosen to give us power to detect twofold changes in gene expression using DESeq2 according to guidelines in Ching et al.^48^

### RNA isolation and RNA-seq

RNA isolation and DNase treatment was performed using the Qiagen RNeasy Mini Kit according to manufacturer’s instructions. RNA integrity was verified using an Agilent Bioanalyzer. Library preparation and RNA-seq was performed by Q2 Solutions (Morrisville, NC). Sequencing libraries were created using the IlluminaTruSeq Stranded mRNA method. Samples were sequenced on an Illumina HiSeq 2500 machine.

### RNA-seq mapping and analysis

Adapter sequence and poor quality bases (*N* or ≤Q7) were clipped from the ends of reads using ea-utils. https://github.com/ExpressionAnalysis/ea-utils. Reads were mapped to the human genome (hg38) using tophat and bowtie 2^49^. Differential expression analysis was performed using DESeq2^50^ using six replicates per condition. The gene model used for DESeq2 was Ensembl GRCh38. After running DESeq2, we excluded genes that were not expressed in any condition (FPKM < 1 in all samples) or did not have an Entrez Gene identifier from further analysis; 11,720 genes remained. We defined differentially expressed genes as those with DESeq2’s adjusted *p*-value (Benjamini–Hochberg correction) <10^−3^ and a log_2_-fold change >1.

We defined genes as bound by FXR in primary human hepatocytes using the ChIP-seq data set of Zhan et al.^51^ (accession GSE57227), thresholding on peaks with score ≥25 in the DMSO or GW4064 treatment condition, and intersecting with Ensembl-defined gene bodies using BEDTools (peaks and genes both hg19). Gene set enrichment analysis was performed using GSEA (software version 2.2.4)^34^, as follows: the GseaPreranked function was applied to the list of all expressed genes (FPKM > 1 in at least one sample) ranked by the log_2_-fold change estimate from DESeq2 using options -collapse = false, -norm = meandiv, -scoring_scheme = weighted, -mode = Max_probe, -nperm = 1000, -set_min = 10. Entrez gene identifiers were used to map between gene expression and gene sets. The KEGG and Reactome gene sets from MSigDB version 6.0 were used for enrichment analysis (*n* = 176 and *n* = 648 gene sets passing size filters, respectively). The FDR value calculated by GSEA’s permutation test was used to determine statistical significance for each gene set (FDR < 5%) and the normalized enrichment score (NES) from GSEA used as a measure of the degree to which a gene set is overrepresented at the top or bottom of the ranked list of genes.

### Immunofluorescence

Spinning disc laser confocal microscopy (Ultraview, Perkin-Elmer) was used to perform high content analysis on hepatocytes immunostained with anti-human FXR/NR1H4 monoclonal antibody (R&D Sytems/Perseus Proteomics, clone A9033A). In brief, cells were fixed with 4% formaldehyde for 20 mins followed by permeabilization with 0.1% Saponin for 20 mins. Cells were incubated overnight at 4 °C with 2 µg mL^−1^ anti-FXR antibody. Goat anti mouse secondary antibody conjugated to an Alexa647 probe was used to detect the primary antibody. Cells were counterstained with Hoechst (2 µg mL^−1^).

### Code availability

We used publicly available software (URLs listed below) in conjunction with the above described algorithms in the sequencing processing pipeline (sections Whole-genome sequencing, Association testing, RNA-seq mapping and analysis).

BWA 0.7.10 mem, https://github.com/lh3/bwa

GenomeAnalysisTKLite 2.3.9, https://github.com/broadgsa/gatk/

SAMtools 1.3, http://samtools.github.io/

BEDTools v2.25.0-76-g5e7c696z, https://github.com/arq5x/bedtools2/

Variant Effect Predictor https://github.com/Ensembl/ensembl-vep

BOLT-LMM https://data.broadinstitute.org/alkesgroup/BOLT-LMM/downloads/

Ea-utils https://github.com/ExpressionAnalysis/ea-utils

Tophat https://ccb.jhu.edu/software/tophat/index.shtml

DESeq2 v1.14.1 http://bioconductor.org/packages/devel/bioc/html/DESeq2.html

GSEA v2.2.4 http://software.broadinstitute.org/gsea/index.jsp

### Data availability

Sequence variants passing GATK filters have been deposited in the European Variation Archive, accession number PRJEB15197. RNA-seq data have been deposited in the Gene Expression Omnibus, accession number GSE102870.

## Electronic supplementary material

Supplementary Information

Description of Additional Supplementary Files

Supplementary Data 1

Supplementary Data 2
