# Supplementary material for: A rare missense variant in NR1H4 associates with lower cholesterol levels
Source: Commun Biol. 2018 Feb 8;1:14. doi: 10.1038/s42003-018-0015-9 (PMC6123719; doi:10.1038/s42003-018-0015-9)
Supplement: Supplementary file 1 — Supplementary Information [file 42003_2018_15_MOESM1_ESM.pdf]

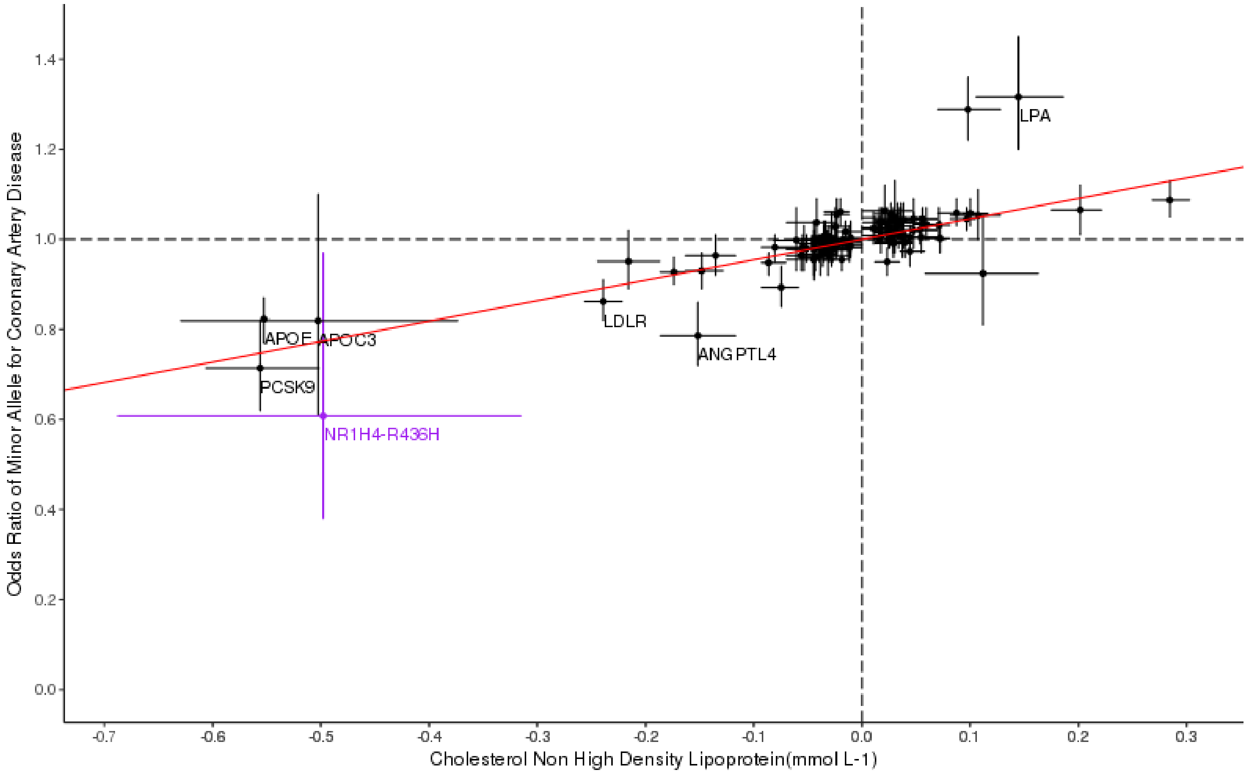

Supplementary Figure 1: Relationship between the effect of sequence variants on non-HDL cholesterol and the risk of coronary artery disease.

Shown are estimated odds ratios of the minor allele of variants for coronary artery disease occurring at or before 75 in Iceland among 25,544 cases and 311,807 controls as a function of the estimated effect of the minor allele on non-HDL cholesterol levels among 136,326 participants for whom data were available. In addition to the effect of *NR1H4* R436H (shown in purple), the effects of 108 variants reported to associate with non-HDL cholesterol levels by Do et al [1] are shown for comparison. For the sequence variants with the largest effects, the affected gene is shown. The error bars represent 95% confidence intervals. The red line indicates the best linear-regression fit through the origin without R436H. To convert the values for non-HDL from mmol L^{-1} to mg dL^{-1} multiply by 38.67.

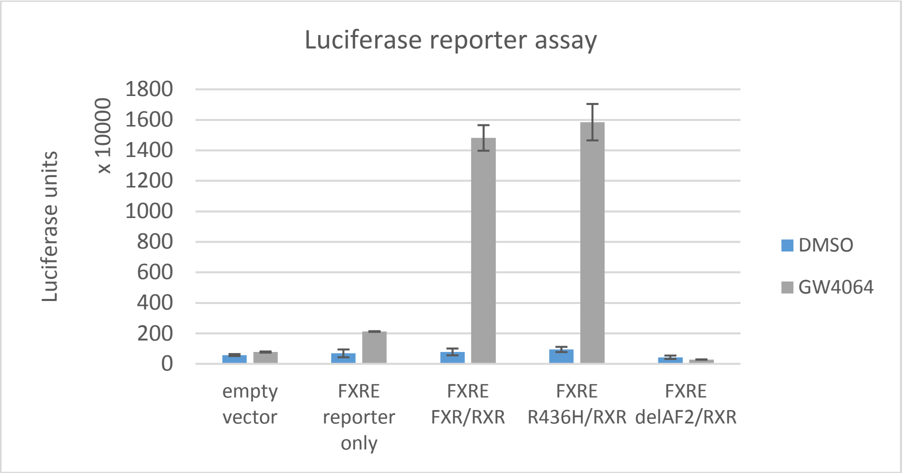

Supplementary Figure 2. Luciferase reporter assays in HepG2 cells show that NR1H4/FXR R436H can activate transcription in response to FXR agonist GW4064.

HepG2 cells were transfected with either 1) empty reporter vector, 2) reporter containing an FXR-response element (FXRE), 3) FXRE reporter and plasmids expressing wild-type FXR and RXR (FXR/RXR), 4) FXRE reporter and plasmids expressing R436H FXR and RXR (R436H/RXR) or 5) FXRE reporter and plasmids expressing non-functional FXR lacking the AF2 domain and RXR (delAF2/RXR). Cells were treated with 5µM GW4064 or DMSO for 24h and luciferase activity assessed as described in the methods. One experiment representative of three independent experiments is shown. For each experiment, transfection and drug treatment were performed in quadruplicate and error bars represent the standard deviation.

A *NR1H4* knockout lines: exon 4 sequence

```
Reference       CAGATTTCCTCGTCATCCTATTATTCCAACCTGGGTTTCTACCCCCAGCAGCCTGAAGAGTGGTACTC-TCCTGGAATATATGAACTCA
KO1-A 11bp del  CAGATTTCCTCGTCATCCTATTATTCCAACCTGGGTTTCTACCCCCAGCAGCCTGAAGAGTGGT------------ATATATGAACTCA
KO1-B 1bp del   CAGATTTCCTCGTCATCCTATTATTCCAACCTGGGTTTCTACCCCCAGCAGCCTGAAGAGTGGTACT--TCCTGGAATATATGAACTCA
KO2-A 8bp del   CAGATTTCCTCGTCATCCTATTATTCCAACCTGGGTTTCTACCCCCAGCAGCCTGAAGAG---------TCCTGGAATATATGAACTCA
KO2-B 1bp ins   CAGATTTCCTCGTCATCCTATTATTCCAACCTGGGTTTCTACCCCCAGCAGCCTGAAGAGTGGTACTCTTCCTGGAATATATGAACTCA
```

B *NR1H4* R436H lines: exon 11 sequence

```
Reference   CCTGTCTCCTGGGTCGCCTGACTGAATTACGGACATTCAATCATCACCACGCTGAGATGCTGATGTCATGGAGAGTAAACGACCACAAG
R436H1-A    CCTGTCTCCTGGGTCACCTCACAGAATTACGGACATTCAATCATCACCACGCTGAGATGCTGATGTCATGGAGAGTAAACGACCACAAG
R436H1-B    CCTGTCTCCTGGGTCACCTCACAGAATTACGGACATTCAATCATCACCACGCTGAGATGCTGATGTCATGGAGAGTAAACGACCACAAG
R436H2-A    CCTGTCTCCTGGGTCACCTCACAGAATTACGGACATTCAATCATCACCACGCTGAGATGCTGATGTCATGGAGAGTAAACGACCACAAG
R436H2-B    CCTGTCTCCTGGGTCACCTCACAGAATTACGGACATTCAATCATCACCACGCTGAGATGCTGATGTCATGGAGAGTAAACGACCACAAG
```

C

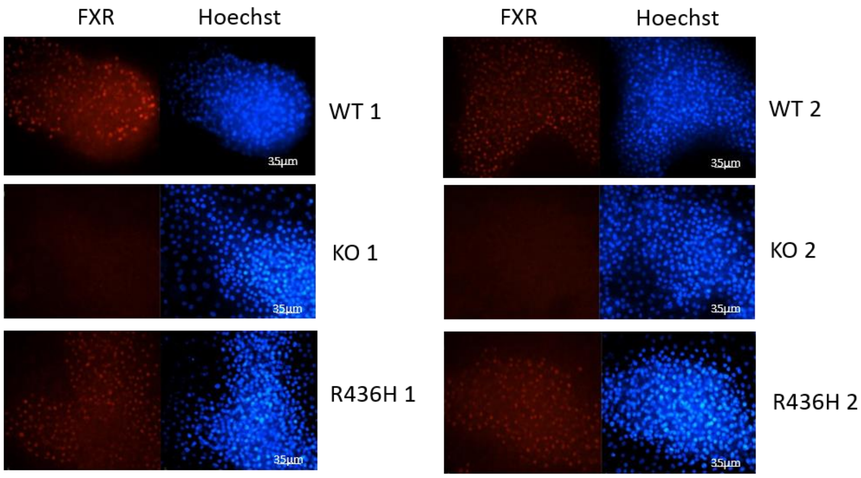

Supplementary Figure 3: Engineering of *NR1H4* mutant iPSC lines with CRISPR-Cas9.

(A) Sequence of the targeted portion of exon 4 in *NR1H4* knockout lines, KO1 and KO2, compared to the reference. (B) Sequence of the targeted portion of exon 11 in *NR1H4* R436H lines, R436H1 and R436H2, compared to the reference. In addition to R436H, silent modifications at codons L437 and T438 were introduced to aid screening. The edited codons are highlighted in red with changed bases underlined. (C) Immunofluorescence for FXR protein in wild-type (WT), *NR1H4* knockout (KO) and R436H iPSC-derived hepatocytes. Nuclei were counterstained with Hoechst.

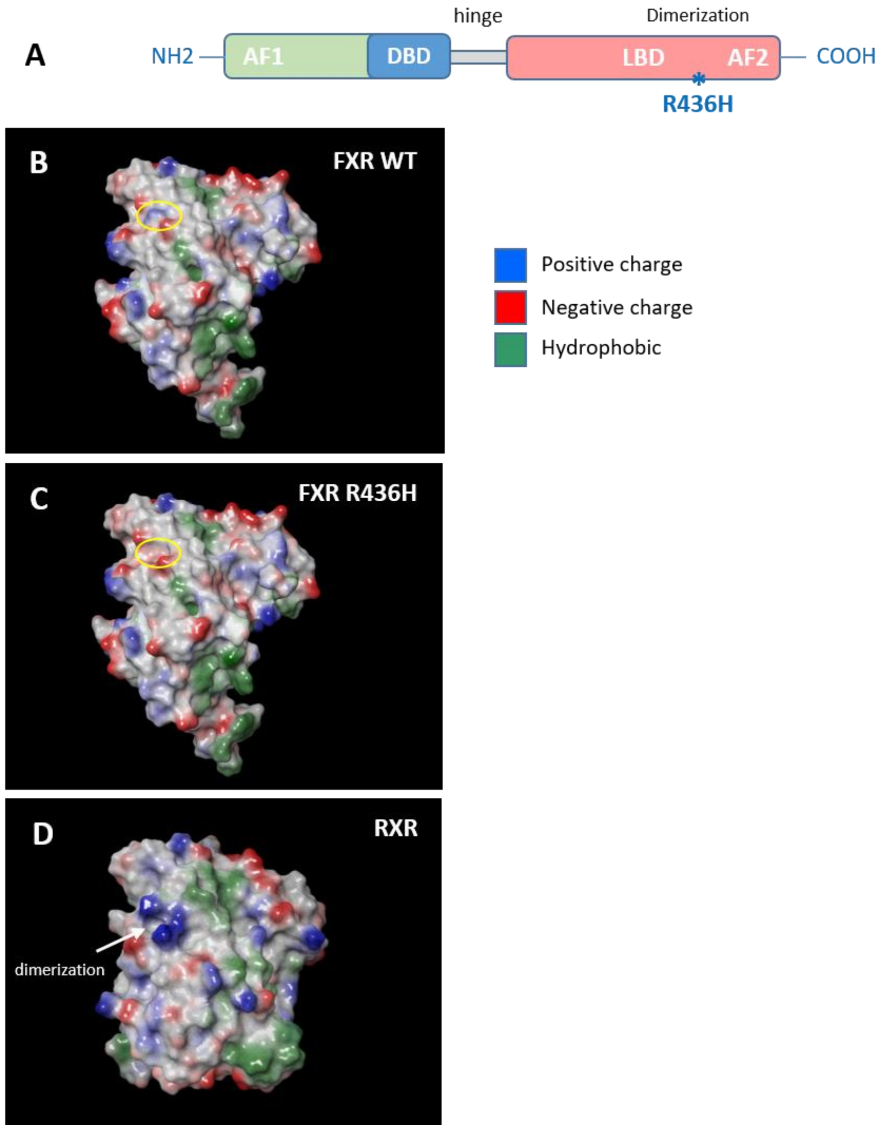

Supplementary Figure 4: Effect of *NR1H4* R436H on FXR protein.

(A) R436H is located on the dimerization interface of FXR distal from the ligand-independent activation domain (AF1), DNA-binding domain (DBD), ligand binding domain (LBD) and ligand-dependent activation domain (AF2). Visualization of the protein surface of wild-type FXR (B), mutant R436H FXR (C) and wild-type RXR (D). The crystal structures of FXR (PDB ID: 4OIV) and RXR (PDB ID: 2P1T) were used. The histidine 436 mutation was modelled using Maestro 11.1 (Schrodinger, New York). All proteins were first prepared using the Protein Preparation Wizard in Maestro; side chains and hydrogen atoms were added, the proper ionization states were assigned, and energy was minimized. Protein surface analysis was performed using the built-in tool from Maestro, where blue represents overall positive charge, red represents overall negative charge, and green represents hydrophobicity. The three proteins are displayed in a superimposed manner. For FXR, the yellow circle highlights amino acid 436, for RXR the arrow points to the dimerization interface. Changing FXR arginine 436 to a histidine, leads to a decrease in surface charge close to residue 436. The dimerization interface of RXR is positively charged so RXR may have increased affinity for the more negatively charged FXR R436H compared to wild-type FXR.

Supplementary Table 1. Lipid measurements used in this study.

The total number of lipid measurements (N measurements) and individuals measured (N individuals measured) used for this study. The total number of individuals with lipid values used in the regression analysis is shown (Total individuals used) as well as the breakdown into those that were chip-typed and directly imputed (Direct imputation) and those that were the first- and second-degree relatives of chip-typed individuals who had their genotypes inferred based on genealogy (Familial imputation).

	**Total cholesterol**	**non-HDL cholesterol**	**LDL cholesterol**	**HDL cholesterol**	**Triglycerides**
**N measurements**	684,535	566,543	460,261	570,893	514,709
**N individuals measured**	159,163	141,149	130,794	141,568	141,138
**Total individuals used**	150,211	136,326	126,220	136,736	119,624
**Direct imputation**	99,812	92,969	87,055	93,166	81,081
**Familial imputation**	50,399	43,357	39,165	43,570	38,543

Supplementary Table 2: *NR1H4* R436H variant

The minor allele frequency (MAF, percentage) in Iceland is shown alongside the estimated frequency of carriers in Iceland. Maximum MAF abroad was obtained from the Genome Aggregation Database (accessed June 21st 2017). Variant effect predictor (Ensembl) was used to annotate the consequence of variants on *NR1H4* transcripts and protein.

**Name**	**Chromosome position (hg38)**	**MAF Iceland**	**Max MAF Abroad**	**Imputation information**	**Allele (min/maj)**	**Gene**	**Transcript change**	**Protein change**
R436H (rs750672942)	chr12:100563377	0.11%<br><br>~1 in 450 carriers	0.01 % (South Asian); absent in Europeans	0.96	A/G	*NR1H4*	NM_005123.3:c. 1307G>A<br>NM_001206977.1:c. 1319G>A<br>NM_001206992.1:c. 1337G>A<br>NM_001206993.1:c. 1349G>A	NP_005114.1: p.Arg436His<br>NP_001193906.1 :p.Arg440His<br>NP_001193921.1 :p.Arg446His<br>NP_001193922.1 :p.Arg450His

Supplementary Table 3: Associations of missense variants in *NR1H4* with total cholesterol levels.

**Chromosome position**	**Coding effect**	**MAF**	**p-value**	**effect**
chr12:100563377	NP_005114.1:p.Arg436His	0.11%	4.21×10^{-10}	-0.47
chr12:100532530	NP_005114.1:p.Met173Thr	1%	0.12	-0.04
chr12:100534946	NP_005114.1:p.His215Tyr	0.27%	0.50	0.033
chr12:100563269	NP_005114.1:p.Asp400Tyr	0.02%	0.54	0.109

Supplementary Table 4: Association of *NR1H4* missense variant with cardiovascular disease in Iceland

The effect on disease risk is shown as an odds ratio (OR) with 95% confidence interval (CI).

**Disease**	**OR (95% CI)**	**p-value**	**N (cases/controls)**
Myocardial infarction early onset	0.02 (0.00, 0.25)	0.0032	3027/332745
Myocardial infarction at or before age 75	0.49 (0.27, 0.87)	0.014	16256/319516
Myocardial infarction	0.68 (0.43, 1.07)	0.092	23965/311807
Coronary artery disease early onset	0.25 (0.07, 0.81)	0.022	5288/334875
Coronary artery disease at or before age 75	0.61 (0.38, 0.97)	0.039	25544/328262
Coronary artery disease all	0.72 (0.48, 1.07)	0.103	37782/318845
Ischemic stroke	0.68 (0.33, 1.40)	0.29	5626/262087
Peripheral artery disease	0.81 (0.34, 1.93)	0.64	3738/292291

Supplementary Table 5: Association of *NR1H4* R436H with hepatobiliary traits

Markers of hepatobiliary function were measured as described in the methods. Effect sizes are shown as a percentage change or in grams per liter (g/L) as well as in standard deviations (SD); values in brackets represent the 95% confidence interval (CI). For markers with an approximately log-normal distribution, population mean and standard deviation were calculated for log-transformed values and then transformed back to original units.

**Variable**	**Effect (95% CI)**		**p-value**	**Value in population**	**N measured**
	**% change**	**SD estimate**			
Alkaline Phosphatase	-1.73 (-4.86, 1.35)	-0.08 (-0.23, 0.07)	0.28	96.8 (60.3, 155.6) U/L	154097
Alanine Transaminase	1.33 (-1.43, 4.06)	0.05 (-0.05, 0.16)	0.34	32 (17.5, 58.2) U/L	172086
Aspartate Transaminase	1.50 (-0.94, 3.91)	0.06 (-0.04, 0.16)	0.22	29.8 (17.2, 51.6) U/L	164467
Bilirubin	-1.18 (-4.62, 2.19)	-0.04 (-0.16, 0.08)	0.50	9.8 (5.0, 19.1) µm/L	109748
Gamma Glutamyl Transpeptidase	2.21 (-2.1, 6.43)	0.06 (-0.05, 0.17)	0.31	32.9 (13.9, 77.7) U/L	156692
	**g/L**	**SD estimate**			
Albumin	-0.14 (-0.87, 0.58)	-0.02 (-0.15, 0.10)	0.71	39.6 (± 5.8) g/L	92163

Supplementary Table 6: Power calculations for R436H variant and hepatobiliary traits

For each phenotype we have 80% power to reject the null hypothesis of no effect given the absolute effect size (in SD or OR) indicated at a two sided α=0.05. Power calculations are described in the methods.

**Phenotype**	**Absolute effect with 80% power**	**Effect unit**
Alanine Transaminase	0.15	SD
Albumin	0.15	SD
Alkaline Phosphatase	0.21	SD
Aspartate Transaminase	0.14	SD
Bilirubin	0.17	SD
Gamma Glutamyl Transpeptidase	0.17	SD
Gallstones	1.73	OR

Supplementary Table 7: *NR1H4* splice donor mutation

The minor allele frequency (MAF, percentage) for the splice donor mutation in Iceland is shown alongside the estimated frequency of carriers in Iceland. Carriers were observed on sequence and the variant reimputed (see methods). Maximum MAF abroad was obtained from the Genome Aggregation Database (accessed June 21st 2017). Variant effect predictor (Ensembl) was used to annotate the consequence of variants on *NR1H4* transcripts and protein.

**Name**	**Chromosome position (hg38)**	**MAF Iceland**	**Max MAF Abroad**	**Imputation info**	**Allele (min/maj)**	**Gene**	**Transcript change**	**Protein change**
splice donor deletion	chr12:100536607	0.016%<br>~1 in 3,000 carriers	Absent abroad	0.99	A/AATTGT	*NR1H4*	NM_005123.3:c.<br>817_819+2delATTGT<br>NM_001206977.1:c.<br>829_831+2delATTGT<br>NM_001206992.1:c.<br>847_849+2delATTGT<br>NM_001206993.1:c.<br>859_861+2delATTGT	

Supplementary Table 8: Associations of the *NR1H4* splice donor mutation with lipid levels and hepatobiliary traits.

Effect sizes and 95% confidence interval (CI) is shown in original units or percentage change as well as in standard deviations (SD). For each measurement, the mean value and standard deviation of the population are given. For traits with a log-normal distribution, these were calculated on transformed values and then transformed back to original units. To convert the values for total, non-HDL, LDL and HDL cholesterol to mg dL^{-1} multiply by 38.67. To convert the values for triglycerides to mg dL^{-1} multiply by 88.57.

**Variable**	**Effect (95% CI)**		**p-value**	**Value in population**
	**mmol L^{-1}**	**SD estimate**		
Total Cholesterol	-0.31 (-0.81, 0.19)	-0.26 (-0.69, 0.16)	0.22	5.33 ± 1.168 mmol L-1
Non-HDL Cholesterol	-0.37 (-0.90, 0.16)	-0.32 (-0.77, 0.14)	0.18	4.948 ± 1.166 mmol L-1
LDL Cholesterol	-0.23 (-0.75, 0.29)	-0.22 (-0.71, 0.28)	0.39	3.327 ± 1.053 mmol L-1
HDL Cholesterol	-0.01 (-0.22, 0.19)	-0.03 (-0.50, 0.44)	0.90	1.425 ± 0.441 mmol L-1
	**% change**	**SD estimate**		
Triglycerides	9.24 (-20.71, 35.52)	0.17 (-0.32, 0.66)	0.50	1.24 (0.73, 2.09) mmol L-1
Alkaline Phosphatase	-2.42 (-11.00, 5.79)	-0.12 (-0.53, 0.29)	0.58	96.8 (60.3, 155.6) U/L
Alanine Transaminase	-1.62 (-8.71, 5.22)	-0.06 (-0.33, 0.21)	0.65	32 (17.5, 58.2) U/L
Aspartate Transaminase	0.18 (-6.62, 6.75)	0.01 (-0.27, 0.29)	0.96	29.8 (17.2, 51.6) U/L
Bilirubin	-1.59 (-12.09, 8.36)	-0.06 (-0.41, 0.30)	0.76	9.8 (5.0, 19.1) µm/L
Gamma Glutamyl Transpeptidase	2.99 (-9.3, 14.57)	0.08 (-0.23, 0.39)	0.62	32.9 (13.9, 77.7) U/L

Supplementary Table 9: Associations of splice donor mutation in *NR1H4* with gallstones in Iceland

The effect on disease risk is shown as an odds ratio (OR) with 95% CI.

**Disease**	**OR (95% CI)**	**p-value**	**Case/Controls (no.)**
Gallstones	2.03 (0.51, 8.04)	0.31	8447/339288

Supplementary Table 10: Power calculations for *NR1H4* splice donor mutation

For each phenotype we have 80% power to reject the null hypothesis of no effect given the absolute effect size (in SD) indicated at a two sided α=0.05. Power calculations are described in the methods.

**Phenotype**	**Absolute effect with 80% power (SD)**
Alanine Transaminase	0.37
Alkaline Phosphatase	0.61
Aspartate Transaminase	0.56
Bilirubin	0.55
Gamma Glutamyl Transpeptidase	0.45
HDL Cholesterol	0.67
LDL Cholesterol	0.72
Non HDL Cholesterol	0.67
Total Cholesterol	0.59
Triglycerides	0.71

Supplementary Table 11. Selected lipid related pathways with significant enrichment for genes upregulated in *NR1H4* R436H agonist treated cells compared to wild-type agonist treated cells (see Supplementary Data 2 for full list). Enrichment was assessed using GSEA. The normalized enrichment score (NES) reflects the degree to which a gene set is overrepresented at the top or bottom of a ranked list of genes, pathways with FDR<5% (calculated from GSEA's permutation test) were considered statistically significant.

**Name**	**R436H agonist vs WT agonist, upregulated genes (NES)**	**R436H agonist vs WT agonist, upregulated genes (FDR)**
**Reactome pathways (674 pathways tested)**		
Sphingolipid Metabolism	2.08	0.004
Phospholipid Metabolism	1.8	0.038
Cholesterol Biosynthesis	1.77	0.047
Metabolism Of Lipids and Lipoproteins	1.76	0.05
**KEGG pathways (186 pathways tested)**		
Steroid Biosynthesis	1.98	0.008
Sphingolipid Metabolism	1.82	0.023
Valine Leucine and Isoleucine Degradation	1.78	0.028

Supplementary Note:

A rare predicted NR1H4 loss-of-function variant in the Icelandic population

Based on an expanded whole genome sequencing dataset of 31,621 Icelanders, we found a 5bp deletion predicted to remove the last amino acid of *NR1H4* exon 7 (of 11 exons) and a consensus splice donor sequence (splice donor deletion; NCBI reference sequence NM_005123.3:c.817_819+2delATTGT) in 12 individuals. This mutation occurs in all protein-coding *NR1H4* transcripts and is predicted to break the wild-type donor splice site by Human Splicing Finder [2]. We confirmed 49 carriers of this variant in Iceland by Sanger sequencing the relatives of carriers on whole genome sequence and imputation into 299,767 Icelanders (MAF = 0.016%; imputation information = 0.99) (Supplementary Table 7). All carriers of this predicted loss-of-function variant in Iceland are heterozygous and the variant has not been reported outside of Iceland (Genome Aggregation Database). The *NR1H4* splice donor mutation is not significantly associated with levels of total cholesterol (effect = -0.31 mmol L^{-1}; 95% CI -0.81 to 0.19 mmol L^{-1}; effect in SD= -0.26; P=0.22) or non-HDL cholesterol levels (effect = -0.37 mmol L^{-1}; 95% CI -0.90 to 0.16 mmol L^{-1}; effect= -0.32 SD; P=0.18) (Supplementary Table 8). However, as this variant is extremely rare, the confidence intervals for these associations are large. We have 80% power to detect a 0.59 SD effect of the mutation on total cholesterol levels (Supplementary Table 10). We found no significant association between the *NR1H4* splice donor mutation and hepatobiliary traits (all $P \geq 0.50$; 80% power to detect effects of 0.37 to 0.61 SD on hepatobiliary markers) (Supplementary Tables 8-10). Thus, while we can't exclude an effect of the mutation on these traits, we do not see evidence for effects of the magnitude observed in patients with biallelic loss-of-function FXR mutations [3]. This suggests, as reported, a recessive inheritance model for cholestatic disease.

Supplementary References

1. Do R*, et al.* Exome sequencing identifies rare LDLR and APOA5 alleles conferring risk for myocardial infarction. *Nature* **518**, 102-106 (2015).

2. Desmet FO, Hamroun D, Lalande M, Collod-Beroud G, Claustres M, Beroud C. Human Splicing Finder: an online bioinformatics tool to predict splicing signals. *Nucleic Acids Res* **37**, e67 (2009).

3. Gomez-Ospina N*, et al.* Mutations in the nuclear bile acid receptor FXR cause progressive familial intrahepatic cholestasis. *Nat Commun* **7**, 10713 (2016)
