# Supplementary material for: A rare missense variant in NR1H4 associates with lower cholesterol levels
Source: Commun Biol. 2018 Feb 8;1:14. doi: 10.1038/s42003-018-0015-9 (PMC6123719; doi:10.1038/s42003-018-0015-9)
Supplement: Supplementary file 2 — Description of Additional Supplementary Files [file 42003_2018_15_MOESM2_ESM.docx]

**Description of Additional Supplementary Files**

File Name: Supplementary Data 1

Description: Full list of gene expression changes in the various conditions. (A) Wild-type cells treated with agonist (trt) vs. untreated cells (untrt). (B) NR1H4 R436H cells treated with agonist vs. untreated cells. (C) NR1H4 knockout cells treated with agonist vs. untreated cells. (D) NR1H4 R436H untreated hepatocytes vs. wild-type untreated hepatocytes (E) NR1H4 R436H agonist treated hepatocytes vs. wild-type agonist treated hepatocytes. (F) NR1H4 knockout agonist treated hepatocytes vs. wild-type agonist treated hepatocytes.

File Name: Supplementary Data 2

Description: Full list of pathways significantly enriched for genes upregulated in NR1H4 R436H agonist-treated cells compared to wild-type agonist-treated cells and in NR1H4 knockout agonist-treated cells compared to wild-type agonist-treated cells. Enrichment was assessed using GSEA, 674 Reactome pathways and 186 KEGG pathways were queried. The normalized enrichment score (NES) reflects the degree to which a gene set is overrepresented at the top or bottom of a ranked list of genes, pathways with FDR<5% (calculated from GSEA’s permutation test) were considered statistically significant.
